# The association between objective tongue color and endoscopic findings: results from the Kyushu and Okinawa population study (KOPS)

**DOI:** 10.1186/s12906-015-0904-0

**Published:** 2015-10-16

**Authors:** Mosaburo Kainuma, Norihiro Furusyo, Yoshihisa Urita, Masaharu Nagata, Takeshi Ihara, Takeshi Oji, Toshiya Nakaguchi, Takao Namiki, Jun Hayashi

**Affiliations:** Community Medicine Education Unit, Graduate School of Medical Sciences, Kyushu University, 3-1-1 Maidashi, Higashi-ku, Fukuoka 812-8582 Japan; Department of General Internal Medicine, Kyushu University Hospital, 3-1-1Maidashi, Higashi-ku, Fukuoka 812-8582 Japan; Department of General Medicine and Emergency Care, Toho University School of Medicine, Omori Hospital, 6-11-1Omori-nishi, Ota-ku, Tokyo 143-8541 Japan; Center for Frontier Medical Engineering, Chiba University, 1-33Yayoicho, Inage-ku, Chiba 263-8522 Japan; Department of Japanese Oriental (Kampo) Medicine, Graduate School of Medicine, Chiba University, 1-8-1Inohana, Chuo-ku, Chiba 260-8670 Japan; Kyushu General Medicine Center, Haradoi Hospital, 6-40-8Aoba, Higashi-ku, Fukuoka 813-8588 Japan

**Keywords:** Tongue color, Endoscopic finding, Helicobacter pylori, Evidenced Based Medicine

## Abstract

**Background:**

The relation between tongue color and gastroesophageal disease is unclear. This study was done to investigate the associations between tongue color (TC), endoscopic findings, *Helicobacter*.pylori infection status, and serological atrophic gastritis (SAG).

**Methods:**

The participants were 896 residents of Ishigaki Island, Okinawa, aged 28–86 years. The tongue was photographed, esophagogastroduodenoscopy was done, and serum antibody to H.pylori was measured. SAG was defined as a serum Pepsinogen (PG)Ilevel ≤70 ng/ml and a PGI/IIratio ≤3.0. TC was measured by the device-independent international commission on Illumination 1976 L*a*b* color space standards at four points: (1) edge, (2) posterior, (3) middle, and (4) apex. We also calculated the ratio of the tongue edge to the three other measured points to examine the association between the coating of the tongue and the endoscopic and laboratory findings.

**Results:**

Participants were excluded who had two or more endoscopic findings (*n =* 315) or who had SAG without seropositivity to *H.pylori* (*n =* 33). The remaining 548 participants were divided into three groups: SAG and seropositive to *H.pylori* (*n =* 67), seropositive to *H.pylori* alone (*n =* 56), and without SAG and seronegative for *H.pylori* (*n =* 425). We divided 425 residents into a single endoscopic finding positive group (*n =* 207) and a negative group, which served as a control (*n =* 218). The most frequent single endoscopic finding was esophageal hernia (*n =* 110), followed by erosive esophagitis (*n =* 35) and erosive gastritis (EG) (*n =* 45). EH was significantly associated with TC (2b*/1b*) (*P <* 0.05). EG was significantly associated with TC (3a*, 3b*) (*P <* 0.05). Seropositivity to *H.*pylori was significantly associated with TC (3 L*, 3 L*/1 L*) (*P <* 0.05, <0.01), and seropositivity to both *H*.pylori and SAG was significantly associated with TC (3 L*/1 L*) (*P <* 0.05). Multivariate analysis extracted TC (3a*, 3b*) as an independent factor associated with a differential diagnosis of EG (Odds ratio (OR) 2.66 *P =* 0.008, OR 2.17 *P =* 0.045).

**Conclusions:**

The tongue body color of the middle area reflects acute change of gastric mucosa, such as erosive gastritis. Tongue diagnosis would be a useful, non-invasive screening tool for EG.

## Background

Although upper gastrointestinal diseases are usually diagnosed by esophagogastroduodenoscopy, it has drawbacks in that there are areas of the world where endoscopes cannot be used and in that there are safety concerns when they are used for screening, including biopsy, as they were shown to have a risk of complication of 0.005 %, from 2003 to 2007 in Japan [1]. Although we think that esophagealgastroduodenoscopy should be done when there is a possibility of positive findings, Arne Faber said that the tongue is a mirror of the stomach [2]. Much has been written about tongue findings in Western internal medicine textbooks, but much remains to be clarified about the significance of tongue findings. Because we felt that there might be a correlation between the tongue and upper gastrointestinal disease and that tongue diagnosis would be useful for screening the disease, we did this study to determine the association of tongue diagnosis and upper gastrointestinal diseases.

Tongue diagnosis is one of the most important diagnostic methods of Kampo medical practice, in which doctors observe tongue color, gloss, shape, and tongue coating in their diagnosis of a patient’s health status [3]. Many studies have reported a correlation between the shape and color of the tongue and an individual’s health [4, 5]. Kampo diagnosis is based on very rich practical experience and the subjective opinions of the physician in the use of this diagnostic method. Therefore, the skills applied in the examination are difficult to understand and quantify. Further, in Japan, although a few small-scale studies have been done on the association between tongue color and endoscopic findings [6–8], none have objectively estimated or made a qualitative diagnosis of the tongue. This represents an obstacle for Kampo medicine to attaining recognition in modern medicine. In order to solve these problems, researchers have developed computer tongue diagnostic support systems that use image processing [9–12]. Furthermore, there have been attempts to match conventional diagnosis of cardiovascular disease, gastric cancer, and rheumatoid arthritis to tongue images that seem to correspond to these diseases [13–16].

However, with these techniques the gloss on the tongue is visible when the image is captured and the system cannot correctly obtain the color of the portion of the tongue masked by the gloss. Recently, we constructed a tongue image analysis system (TIAS) that can be used for computer-aided tongue diagnosis based on tongue color [17, 18]. The key characteristic of the tongue imaging method in TIAS is the exclusion of the influence of external light by use of an integrating sphere to achieve an evenly distributed light intensity with a halogen light source. Further, TIAS can remove the gloss of the tongue surface from its images in order to stabilize the color of the tongue surface and the coating of the tongue.

The purpose of this large-scale study was to use TIAS to objectively investigate the associations between tongue color, endoscopic findings, *H.pylori* infection status, and serological atrophic gastritis.

## Methods

### Study population and study protocol

The current study began in 2007 as a survey of the incidence of vascular events associated with lifestyle-related disease among the general population as a part of the Kyushu and Okinawa Population Study (KOPS) [19–22]. Our study was conducted with residents of Ishigaki City, Okinawa Prefecture who participated in a program of esopahagogastroduodenoscope screening for gastric cancer between October 2012 and January 2013. In the study protocol, after giving informed consent, the tongue was photographed. The photography was conducted in a shady controlled room. The subject’s face was fixed with a chin rest and a forehead rest. As the mouth cannot be opened when both chin and forehead are fixed, first the chin is placed on the chin rest and, after swallowing saliva, the mouth is opened and the tongue extended, following which the forehead is placed against the forehead rest. Each tongue extension is for 20s, and images are taken every 100 ms, for a total of 200 images. After that the operator visually confirmed the tongue color. Secondly, esophagogastroduodenoscopy was done, blood was taken to check for serum antibody to *H.pylori* (anti*-H.pylori*), and serum pepsinogen (PG) I /II and gastrin were measured. We then estimated the associations between tongue color, the endoscopic findings, *H.pylori* infection status, and serological atrophic gastritis. Tongue color was measured by the device-independent international commission on Illumination (CIE) 1976 L*a*b* color space standards at four points: (1) tongue edge, (2) tongue posterior, (3) tongue middle, and (4) tongue apex. The coating of the tongue does not grow on the edge of the tongue, and the color of that point can be considered the color of the tongue body. In contrast, at the other three points the color is a mixture of the coating and the body of the tongue.

**Fig. 1:**
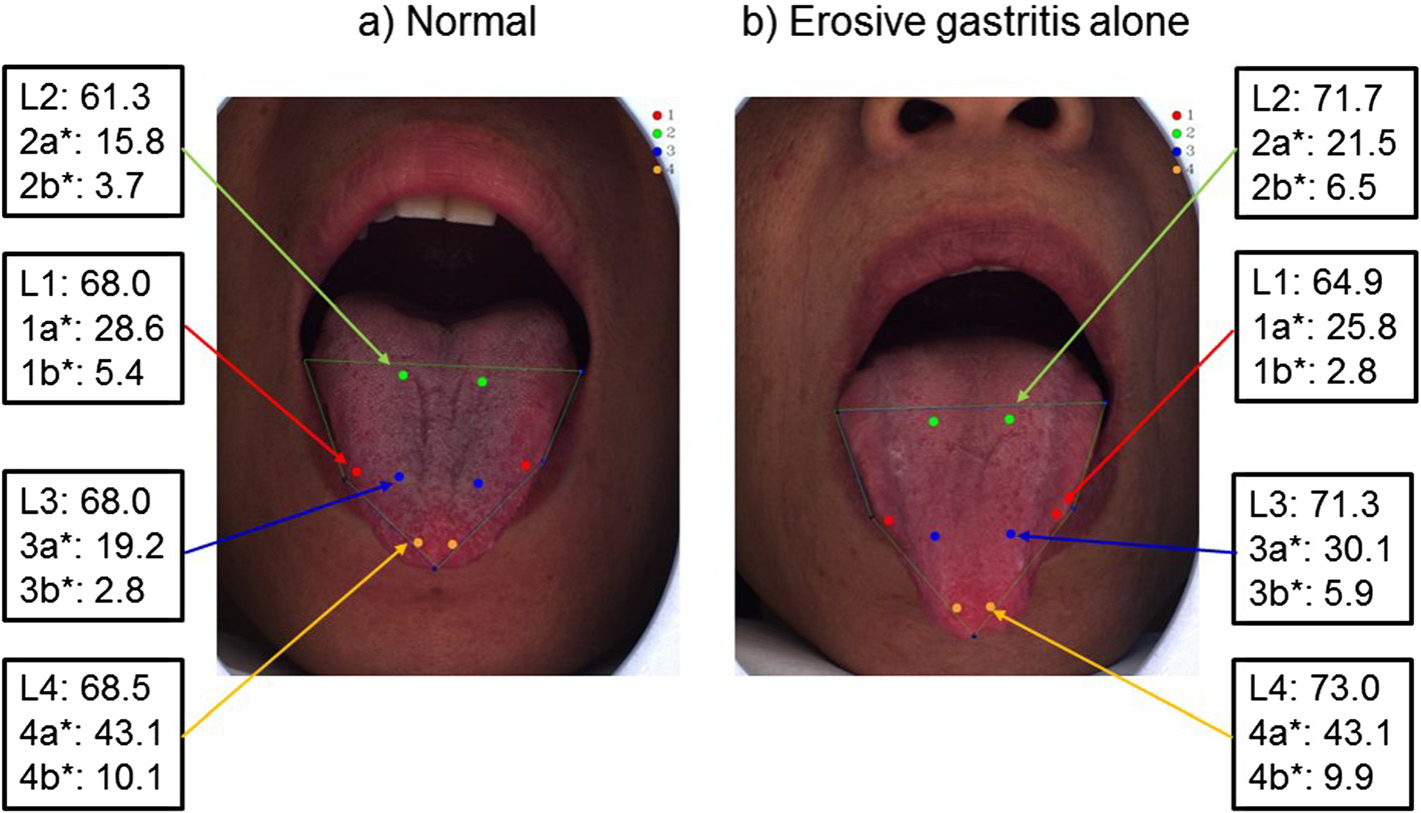
**a**) Example of tongue color from the normal group. Red indicates the tongue edge, green the posterior, blue the middle, and orange the apex. **b**) Example of tongue color with erosive gastritis only

From the point of view of Kampo medicine, it is said that the posterior of the tongue reflects kidney function (traditional medicine), the middle of the tongue reflects the stomach and pancreas (GI tract traditional medicine), and the apex of the tongue reflects the heart (traditional medicine). Therefore, we used these areas to assess the association of tongue color with endoscopic findings. Fig. 1. shows an example of tongue color imaging. The value for this patient with erosive gastritis (3a*, 3b*) is higher than that of the normal control.

In addition, we calculated the ratio of the tongue edge to the other three points measured to examine the association between the coating of the tongue and the endoscopic and laboratory findings. By calculating the ratio to the tongue edge we can confirm that the color of the tongue reflects the body of the tongue, not the coating.

Esophagogastroduodenoscopy was done for 919 residents, and the data of 896 residents from whom we were able to get consent for the tongue color and blood test (age range 28–86 years; mean age 57.7 years; 390 men and 506 women) was available for analysis.

To ensure the validity of the data, all physicians participating in the study were staff members of the Department of General Internal Medicine, Kyushu University Hospital or the Department of General Medicine and Emergency Care, Toho University School of Medicine Omori Hospital. All were trained with regard to the study protocol and the medical procedures necessary for the study. The study protocol was approved by the Ishigaki City Health Center and the Kyushu University Hospital Ethics Committee. Written informed consent was obtained from all participants prior to the examination. Consent to publish the images used was obtained from both of the patients featured. The study was conducted in accordance with the principles of the Helsinki Declaration of 1975, as revised in 2000.

### Tongue image analyzing system (TIAS)

**Fig. 2:**
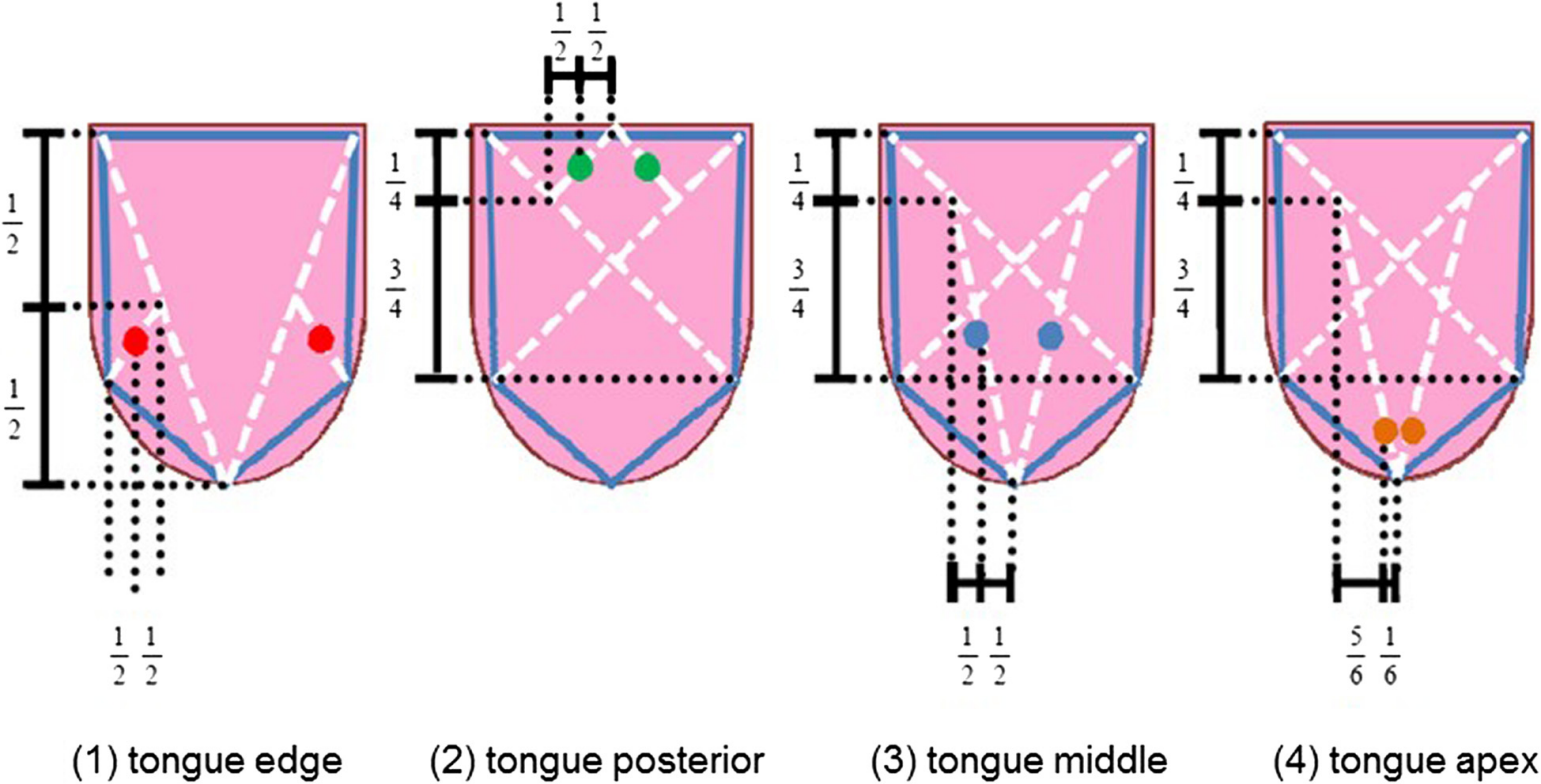
We defined the position of the four points by a ratio calculated by manually specifying five points that define the shape of the tongue

**Fig. 3:**
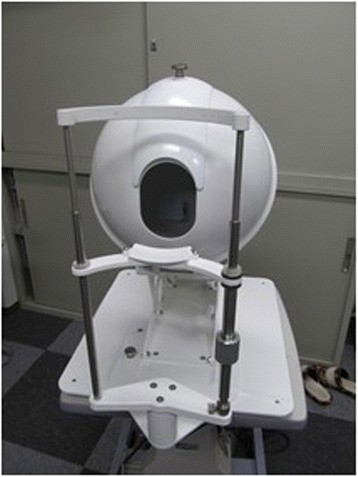
A photograph taken by the Tongue Image Analyzing System (TIAS)

We previously reported on the functionality of the TIAS system, which is equipped with a diffused light source for recording the state of the tongue surface [17, 18, 23]. In brief, when doing photography using TIAS (film image was 1280 × 1024-pixels), calibration of the camera and light source is performed only once, when the power is turned on. When photographing the subject using TIAS, many tongue photographs are taken, from which one image is selected manually for tongue color analysis. We defined the position of the four points by a ratio calculated by manually specifying five points that define the shape of the tongue. The ratio used to determine the four measurement points is illustrated in Fig. 2 The size of the measured tongue area was two 5 mm diameter circles and the measurement of color value is calculated by the average of the two circles. The RGB values at each point are then converted to CIE1976 L*a*b* color space, which is device independent and is designed to be perceptually uniform. This means that a change of the same amount in the L*, a*, or b* value should produce a change of the same visual importance. A photograph taken with TIAS is shown in Fig. 3.

### Esophagogastroduodenoscopy

Each participant underwent esophagastroduodenoscopy at the Ishigaki City Health Center by highly experienced endoscopists who performed each examination without knowledge of the serological data. The endoscopic examination was for esophageal hernia (EH), erosive esophagitis (EE), erosive gastritis (EG), superficial gastritis (SG), gastric ulcer (GU), gastric cancer (GC), erosive duodenitis (ED), duodenal ulcer (DU), and duodenal cancer (DC). EE was defined according to the Los Angeles classifications A-D [24]. GC was diagnosed based on the histological finding and the stage classification and was determined through an evaluation of the clinical examination.

### Testing for antibody to H.pylori

The blood samples of all participants were separated and stored at −80 °C until testing.

The serum IgG level of HP was measured by a commercially available direct ELISA kit (“E Plate ‘Eiken’ HP Antibody” Eiken Kagaku). This ELISA kit was developed in Japan and uses antigen extracted from a domestic strain. It is commonly used in medical studies [25]. Positivity for HP infection was defined as an anti-HP IgG antibody level greater than 10 U/mL in serum.

### Serum PG and gastrin measurement

Serum PG isozymes I and II were measured by a competitive-binding double-antibody radioimmunoassay (PGI/PGIIRIA-BEAD, Abbott Japan Co., Ltd., Tokyo, Japan). The serum gastrin level was measured using an RIA kit (Dinabot Co., Tokyo, Japan). Serological atrophic gastritis was defined based on the results of a serum PGIlevel ≤70 ng/ml and a PGI/IIratio ≤3.0. The assay has a sensitivity of 70.5 % and a specificity of 97.0 % for histological atrophic gastritis [26, 27]. Furthermore, we analyzed the serum PGIlevel as a marker gastric acid secretion [28].

### Statistical analysis

Data are expressed as number (%), mean ± SD, or median with quartiles [25 % - 75 %]. Participant characteristics and the tongue color calculated from tongue photography were compared between participants with and without endoscopic findings, by *H*.pylori status, and by SAG using Fisher’s exact test for categorical variables and unpaired t test or Mann–Whitney test for continuous variables. Variables with a difference of *P <* 0.1 in the univariate analysis were used in multivariate analysis to determine independent, significant predictors. Odds ratios (OR) and 95 % Confident index (CI) were calculated from the multiple logistic regression model after adjustment with each variable. All statistical analyses were performed on a personal computer with the statistical package SPSS 18.0 for windows.

## Results

### Classification by endoscopic findings and anti-H.pylori status (Fig. 4)

**Fig. 4:**
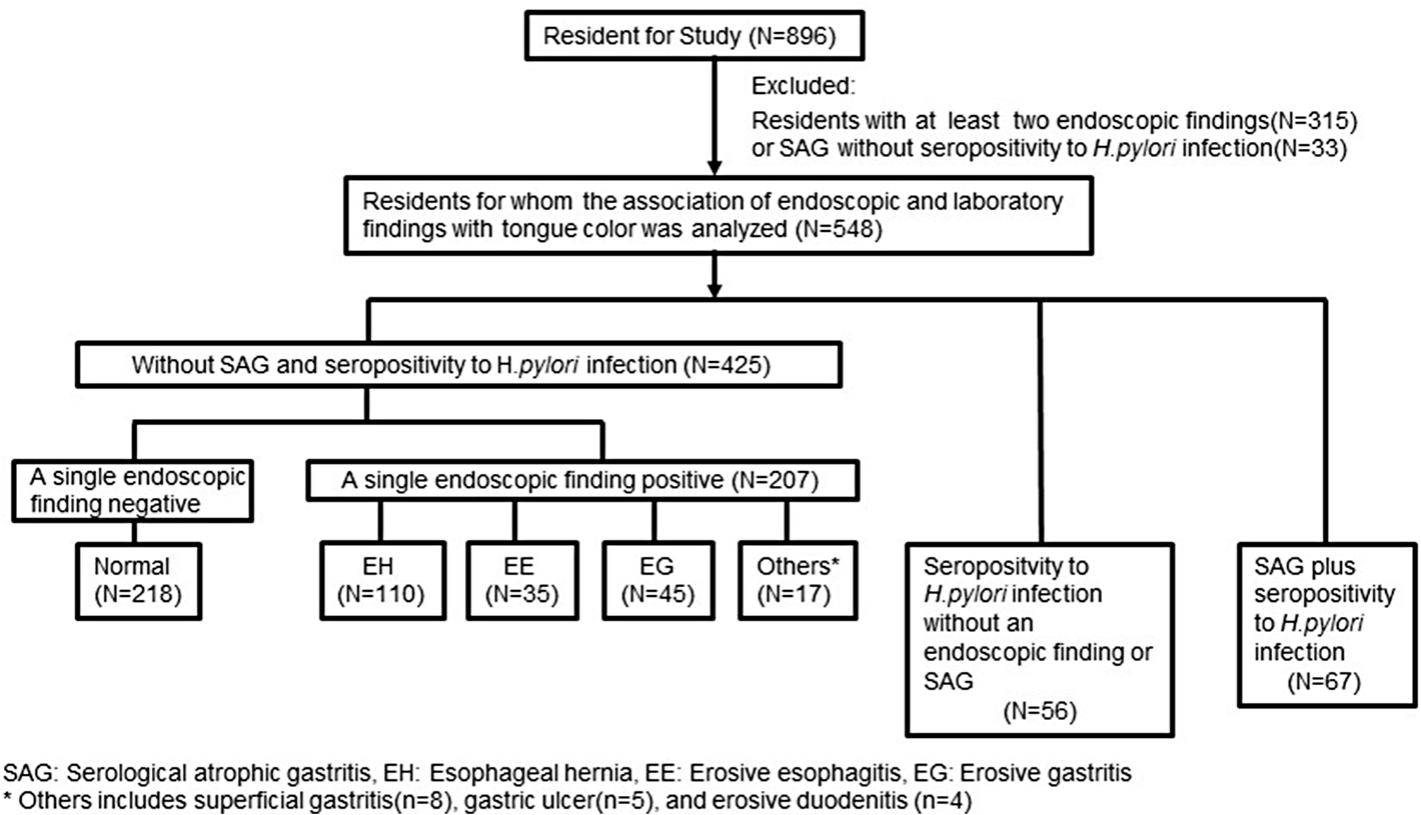
Classification by endoscopic findings, serological atrophic gastritis, and ant-H.pylori status

In this study, we excluded participants who had two or more endoscopic findings (*n =* 315) or who had SAG but were *H.pylori* infection negative (*n =* 33) because we wanted to know the association with each single endoscopic finding and the tongue color and because the discrepancy might have been the result of a false positive SAG result, leaving the data of 548 residents available for analysis. From the serological test, these participants were divided into three groups: SAG and seropositive to *H.pylori* (*n =* 67), seropositive to *H.pylori* alone (*n =* 56), and both SAG and *H.pylori* negative (*n =* 425). The group both SAG and *H.pylori* negative was further divided into a single endoscopic finding positive group (*n =* 207) and a negative group, which served as a control (*n =* 218). The single endoscopic finding positive group was divided as follows: EH (*n =* 110), EE (*n =* 35), EG (*n =* 45), and Others (*n =* 17), which includes SG (*n =* 8), GU (*n =* 5), and ED (*n =* 4).

### The association of endoscopic and laboratory findings with background factors (Table 1)

**Table 1:** The association of endoscopic and laboratory findings factors

	Normal	EH	EE	EG	H.pylori(+)	H.pylori (+) + SAG
	(n = 218)	(n = 110)	(n = 35)	(n = 45)	(n = 56)	(n = 67)
Age (years)	54.5 ± 12.0	57.3 ± 11.9	58.2 ± 10.9	56.3 ± 11.2	58.2 ± 10.5#	60.9 ± 10.9*
Male (%)	61(28.0)	48(43.6)#	20(57.1)*	14(31.1)	18(32.1)	26(38.9)
BMI(Kg/m^2^)	23.2 ± 3.5	24.6 ± 3.7^$^	24.4 ± 4.9	23.3 ± 4.0	23.6 ± 3.6	23.5 ± 3.3
Smoking (%)	9(4.1)	8(7.3)	4(11.4)	1(2.2)	6(10.7)	4(6.0)
Pepsinogen Ι (ng/mL)	45.8 ± 22.2	49.8 ± 30.0	49.1 ± 16.2	51.5±47.8	76.1±29.8*	44.9±16.7
Gastrin (μg/L)	170 ± 311	140 ± 145	146 ± 103	167 ± 179	174 ± 263	200 ± 297

*EH* Esophageal, *EE* Erosiveesophaitis, *EG* Erosive gastitis, *SAG* Serological atrophic gastritis, *H.pylori(+)* ant-Hpylori antibody positive # <0.05 vs Normal, $ < 0.01 vs NOrmal, * < 0.001 vsNormal

EH was significantly associated with sex and BMI (*P <* 0.05, *P <* 0.01). EE was significantly associated with sex (*P <* 0.001). Seropositivity to *H*.pylori was significantly associated with age, PG I, and gastrin (*P <* 0.05, <0.001). Seropositivity to *H*.pylori and SAG were significantly associated with age (*P <* 0.001).

### The association of endoscopic and laboratory findings and tongue color (Table 2)

**Table 2:** The association of endoscopic and laboratory findings to tongue color

	Normal (n = 218)	EH (n = 110)	EE (n = 35)	EG (n = 45)	H.pylori (+) (n = 56)	H.pylori (+) + SAG (n = 67)
1L*	68.0 ± 3.7	67.7 ± 4.2	68.7 ± 4.3	68.6 ± 3.4	68.0 ± 4.0	67.4 ± 3.7
1a*	24.8 ± 4.3	25.6 ± 5.1	25.5 ± 4.1	25.9 ± 5.2	24.6 ± 4.3	24.7 ± 5.0
1b*	5.19 ± 2.91	4.72 ± 2.73	5.14 ± 2.67	6.04 ± 2.75	5.04 ± 3.21	4.93 ± 2.89
2L*	65.4 ± 9.7	64.5 ± 10.7	67.3 ± 5.7	67.1 ± 5.3	65.9 ± 7.9	65.8 ± 6.9
2a*	15.2 ± 5.0	15.3 ± 5.4	14.8 ± 4.5	16.7 ± 3.8	15.5 ± 4.4	15.8 ± 4.9
2b*	5.86 ± 2.97	6.49 ± 7.82	6.09 ± 4.33	6.34 ± 2.74	5.23 ± 2.65	5.07 ± 3.23
3L*	69.4 ± 3.6	69.3 ± 3.5	70.3 ± 3.6	70.4 ± 2.8	70.8 ± 3.2*	69.8 ± 3.7
3a*	23.5 ± 4.2	24.0 ± 4.7	23.9 ± 4.8	25.3 ± 4.2^#^	23.8 ± 6.1	23.4 ± 4.8
3b*	4.72 ± 2.64	4.61 ± 2.35	4.55 ± 2.62	5.81 ± 2.67*	5.04 ± 3.56	4.74 ± 2.65
4L*	66.9 ± 3.8	66.9 ± 3.8	66.6 ± 3.8	67.0 ± 3.1	67.2 ± 3.4	66.2 ± 3.5
4a*	29.7 ± 5.3	30.7 ± 7.0	29.3 ± 4.6	31.0 ± 5.6	29.0 ± 6.9	29.1 ± 4.7
4b*	5.77 ± 2.84	5.32 ± 2.86	5.26 ± 2.17	6.20 ± 3.01	5.27 ± 4.21	5.33 ± 2.88
2L*/1 L*	0.96 ± 0.14	0.96 ± 0.16	0.98 ± 0.09	0.98 ± 0.08	0.97 ± 0.11	0.98 ± 0.11
2a*/1a*	0.62 ± 0.20	0.61 ± 0.20	0.59 ± 0.17	0.66 ± 0.17	0.63 ± 0.14	0.64 ± 0.16
2b*/1b*	0.99 ± 2.61	1.70 ± 3.43^#^	1.63 ± 3.15	0.91 ± 1.94	0.44 ± 4.22	0.78 ± 3.00
3L*/1 L*	1.02 ± 0.05	1.03 ± 0.01	1.02 ± 0.05	1.03 ± 0.04	1.04 ± 0.05^s^	1.04 ± 0.07*
3a*/1a*	0.96 ± 0.16	0.95 ± 0.17	0.95 ± 0.16	1.00 ± 0.16	0.97 ± 0.17	0.96 ± 0.18
3b*/1b*	0.82 ± 1.45	0.81 ± 0.64	0.90 ± 0.77	0.95 ± 0.41	0.44 ± 3.70	1.13 ± 0.97
4L*/1 L*	0.98 ± 0.05	0.98 ± 0.05	0.97 ± 0.05	0.98 ± 0.05	0.99 ± 0.04	0.98 ± 0.06
4a*/1a*	1.21 ± 0.22	1.21 ± 0.22	1.17 ± 0.18	1.22 ± 0.20	1.19 ± 0.26	1.21 ± 0.25
4b*/1b*	1.06 ± 1.24	1.11 ± 1.73	1.11 ± 0.88	0.77 ± 2.24	1.03 ± 0.78	1.08 ± 1.61

*EH* esophageal, *EE* Erosive esophagitis, *EG* Erosive gastritis, *SAG* Serological atrophic gastritis, H.pylori(+) ant-H.pylori antiody positive # < 0.05 vsNormal, $ < 0.01 vs Normal

*refers to international commission on Illumination (CIE) 1976L*a*b* color space standards

EH was significantly associated with TC (2b*/1b*) (*P <* 0.05). EG was significantly associated with TC (3a*, 3b*) (*P <* 0.05). Seropositivity to *H.*pylori was significantly associated with TC (3 L*, 3 L*/1 L*) (*P <* 0.05, <0.01), and seropositivity to both *H*.pylori and SAG was significantly associated with TC (3 L*/1 L*) (*P <* 0.05).

### Analysis of positive predictors of endoscopic and laboratory findings (Table 3)

**Table 3:** Predictors for erosive gastritis

Variables	Univariate analysis		Multivariate analysis	
	OR (95 % Cl)	*P* values	OR (95 % Cl)	*P* values
Age ≥ 65 years	0.75 (0.61-3.27)	0.487	0.63 (0.69-4.12)	0.297
Male	1.16 (0.44-1.77)	0.672	2.04 (0.22-1.11)	0.084
BMI ≥ 25 kg/m^2^	0.94 (0.52-2.33)	0.873	1.10 (0.41-2.10)	0.812
3a* ≥ 25.5	2.69 (0.19-0.71)	0.003	2.66 (0.18-0.77)	0.008
3b* ≥ 5.5	2.59 (0.20-0.74)	0.005	2.17 (0.21-0.97)	0.045

*Cl* confidence interval, *BMI* body mass index

To determine the relative predictive influence of the background factors and endoscopic and laboratory findings, univariate followed by multivariate analysis was done. A significant association with TC was found only for EG. Univariate analysis extracted 3a* and 3b* as significantly associated with a differential diagnosis of EG, and both were extracted in the multivariate analysis (OR 2.66 *P =* 0.008, OR2.17 *P =* 0.045).

## Discussion

This is the first report to demonstrate objective criterion by which TC can be used for the screening of EG. To clarify the relationships and develop an objective system, we constructed the TIAS system with which we can take photographs of the tongue for computerized analysis [17, 18]. Using TIAS, we examined the associations between tongue color and the endoscopic findings of gastric cancer screening.

Previous studies reported that obesity is associated with EH and EE and that H.pylori infection is associated with PGI [29, 30]. Our data supported the findings that obesity is associated with EH and that H.pylori infection is associated with PGI. However, there was no relation between obesity and EE in our analysis of single endoscopic findings. However, when the residents were divided into EE and control groups, the analysis showed a significant correlation between obesity and EE (data not shown).

The results of this large-scale study demonstrated that the analysis of TC by TIAS can be useful in the diagnosis of gastroesophageal diseases. Previous studies reported that the tongue coating becomes more yellowish as gastric erosion becomes more severe [6–8]. Although our study shows that TC (3a*, 3b*) is predictive of EG, the values for 3a*/1a* and 3b*/1b* were near one. Therefore, our data demonstrated that the TC of the body is more useful than the TC of the coating for predicting EG on a large scale. We feel that a tongue body color near red and yellow is predictive of EG. Previous data has shown an association of the coating of the tongue with erosive gastritis. Our results indicate a relevant change in the TC of the body of the tongue. In the tongue diagnosis of Kampo medicine, it is said that the middle of the tongue reflects the function of the GI tract, which was supported by our data. Moreover, a red tongue color is said to reflect fever when there is an inflammatory change. Our data confirmed this tongue diagnosis because the tongue color reflected EG, which supports the middle of the tongue reflecting acute inflammatory change of the GI tract, however, there was no association between EE and TC.

Our results also indicated that EH might be related to the color of the tongue coating, because the value 2b*/1b* had a significantly higher association with EH in comparison with the control, however TC 2b* was not significantly related to EH. The posterior of the tongue is the part nearest to the esophagus, stomach, and duodenum. Therefore, a change in the tongue coating was seen at the posterior of the tongue. It was reported that pH changes leading to erosive mucosal lesions in the esophagus can affect structures in the oral cavity [31]. Thus, the change of pH related to gastroesphageal disease might influence the change of the TC.

Our data showed that there was an association between seropositivity to *H*.pylori and TC L*. L* indicates the brightness of the tongue, which may have been influenced by the way of sticking out the tongue or by the condition of the lighting, making this relation of little use to this study. How to designate the proper way of sticking out the tongue and of controlling the lighting to more accurately determine the L* value are problems that remain to be solved.

It was reported in a series of tongue examinations that pyogenic liver abscess is closely correlated with the progression of diabetes [32]. We also experienced TC change during treatment with a combination of PEG-interferon and ribavirin for chronic hepatitis C patients (data not shown). From these data, we felt that tongue diagnosis might be useful for the follow up of gastritis, not only for the screening of which patients would most benefit from endoscopy.

A limitation of this study is that factors other than gastric disease might affect the TC. Another limitation is that it was conducted with gastric cancer screening and only a small number of residents had endoscopic findings of SG, GU, DU, or ED, thus we could not adequately compare the endoscopic findings for these diseases with the control group. Although the tongue, esophagus, stomach, and duodenum are all parts of the digestive tract, the relation between the tongue and the other parts are unclear, and further studies are needed to clarify the relationships.

## Conclusion

In conclusion, the color of the middle area of the tongue body reflects acute change of gastric mucosa, such as erosive gastritis. Tongue diagnosis would be a useful, non-invasive screening tool for erosive gastritis.

## Abbreviations

CIE: commission on Illumination
DC: Duodenal cancer
DU: Duodenal ulcer
GC: Gastric cancer
GU: Gastric ulcer
ED: Erosive duodenitis
EE: Erosive esophagitis
EG: Erosive gastiritis
EH: Esophageal hernia
H.pylori (+): ant-H.pylori antibody positive
PG: Pepsinogen
SAG: serological atrophic gastritis
SG: Superficial gastritis
TC: tongue color
TIAS: tongue imaging analysis system
